# Update to U.S. Selected Practice Recommendations for Contraceptive Use: Self-Administration of Subcutaneous Depot Medroxyprogesterone Acetate

**DOI:** 10.15585/mmwr.mm7020a2

**Published:** 2021-05-21

**Authors:** Kathryn M. Curtis, Antoinette Nguyen, Jennifer A. Reeves, Elizabeth A. Clark, Suzanne G. Folger, Maura K. Whiteman

**Affiliations:** 1 Division of Reproductive Health, National Center for Chronic Disease Prevention and Health Promotion, CDC.

U.S. Selected Practice Recommendations for Contraceptive Use (U.S. SPR), adapted by CDC from global guidance developed by the World Health Organization (WHO), provides evidence-based guidance on contraceptive use for U.S. health care providers (*1*). During January–February, 2021, CDC evaluated the 2019 WHO recommendation on self-administered subcutaneous depot medroxyprogesterone acetate (DMPA-SC) (*2*). CDC adopted the WHO recommendation on the basis of moderate-certainty evidence that self-administered DMPA-SC is safe and effective, and has higher continuation rates compared with provider-administered DMPA. The new U.S. SPR recommendation states that self-administered DMPA-SC should be made available as an additional approach to deliver injectable contraception. Provider-administered DMPA should remain available. Self-administered DMPA-SC is a user-controlled method that has the potential to improve contraceptive access and increase reproductive autonomy. Self-administered DMPA-SC should be offered in a noncoercive manner through a shared decision-making process between patients and their health care providers, with a focus on patient preferences and equitable access to the full range of contraceptive methods.

## Background

DMPA-SC is a progestin-only injectable contraception method approved by the Food and Drug Administration (FDA) that is similar to intramuscular DMPA (DMPA-IM), but delivered subcutaneously.* Recommendations regarding eligibility and provision of DMPA-SC and DMPA-IM are included in U.S. Medical Eligibility Criteria for Contraceptive Use (U.S. MEC) and U.S. SPR and are the same for both formulations (*1*,*3*). During 2017–2019, 2% of U.S. women aged 15–49 years used DMPA (IM or SC) for contraception; use was most common in younger women (aged 15–24 years), non-Hispanic Black women, and women with lower income.^†^^,^^§^ Because DMPA-SC is administered subcutaneously, the approach lends itself to self-injection.^¶^ Results from U.S. and international studies indicate that self-administered DMPA-SC improves contraceptive continuation rates and has rates of pregnancy, side effects, and adverse events equivalent to those associated with provider administration (*4*).

As part of 2019 guidance on self-care interventions for sexual and reproductive health and rights, WHO recommended that self-administered injectable contraception should be made available as an additional approach to deliver injectable contraception to persons of reproductive age (*2*). The guidance takes into consideration that persons can access information to guide their decisions, make use of appropriate technologies, and seek health services and professional help when necessary (*2*). This approach is consistent with a shared decision-making model to meet patients’ pregnancy planning and reproductive health needs, with a focus on patient preferences and access to the full range of contraceptive methods to minimize risk for coercion** (*5*). Because of the WHO recommendation, CDC initiated a process to determine whether to add a recommendation about self-administration of DMPA-SC to update the U.S. SPR.

## Methods

CDC considered several factors in determining whether to adopt or adapt the WHO recommendation on self-administered DMPA-SC. CDC evaluated a 2019 systematic review (*4*) that contributed to the WHO recommendation. The review assessed the question “Should self-administration be made available as an additional approach to deliver injectable contraception?” and included randomized clinical trials (RCTs) and observational studies comparing self-administered DMPA-SC with provider-administered DMPA-SC or DMPA-IM.^††^ Outcomes included pregnancy; side effects or adverse events; initial use of injectable contraception; continuation rate of injectable contraception; self-efficacy, knowledge, and empowerment; and social harms. Risk of bias was evaluated using The Cochrane Collaboration tool for RCTs and the Evidence Project tool for observational studies. CDC updated the search to identify additional articles published through January 15, 2021, using the same search strategy and inclusion criteria as the 2019 published review. CDC also considered global information on values and preferences about self-administration of DMPA-SC (*2*), and information on implementation of self-administered DMPA-SC in the United States (*6*–*8*).

CDC invited 18 external experts to serve as ad hoc reviewers of the evidence and the WHO recommendation. These reviewers were selected because of their knowledge of methods of contraception and experience in providing family planning services in various settings and to a range of patient populations, including adolescents and persons with disabilities. The reviewers joined one of three teleconferences held in January and February 2021, during which CDC presented the evidence, the WHO recommendation process and outcome, and information about implementing self-administered DMPA-SC in the United States. Participants provided their individual perspectives and experiences about how the evidence might influence U.S. clinical practice and how the WHO recommendation is applicable to the U.S. context. The teleconferences were designed to exchange information; participants were not asked to develop recommendations or a consensus opinion. After the teleconferences, CDC developed the recommendation described in this report, taking into consideration the evidence, the WHO recommendation, and the individual perspectives provided by the expert reviewers.

## Evidence and Rationale

The 2019 systematic review identified six studies that assessed self-administered DMPA-SC compared with provider-administered DMPA-SC or DMPA-IM (*4*). Studies included participants of different age ranges; five studies included participants aged ≥18 years and one study included participants aged ≥15 years. Two of the studies were conducted in the United States. Three of the studies were RCTs and three were prospective cohort studies; all of the studies followed participants for 12 months. Higher rates of continuation were observed with self-administered DMPA-SC than with provider-administered DMPA (SC or IM) (metaanalysis pooled relative risk [RR] = 1.27, 95% confidence interval [CI] = 1.16–1.39 for RCTs and pooled RR = 1.18, 95% CI = 1.10–1.26 for observational studies). Pregnancy was measured in four studies; rates were low overall (≤1%) and did not differ between self-administered and provider-administered groups. Two studies found higher rates of injection site reactions with self-administered DMPA-SC compared with provider-administered DMPA-IM, and two studies found no differences. No other side effects or adverse events were increased with self-administration. None of the studies reported on self-efficacy, knowledge, and empowerment, or social harms. CDC identified one additional secondary analysis from a primary study included in the 2019 published systematic review; this analysis found similar 12-month continuation rates for self-administered DMPA-SC among younger (aged 18–24 years) and older (aged ≥25 years) participants (*9*).

WHO determined the evidence to be of moderate-certainty and that the benefits of self-administration outweighed any potential harms, resulting in a strong recommendation that self-administered injectable contraception should be made available as an additional approach to deliver injectable contraception to persons of reproductive age (*2*). WHO also considered resources, feasibility, equity and human rights, and the potential for the intervention to improve health equity if implemented in the context of an enabling environment (*2*). Implementation issues, such as safe disposal of self-injection equipment, were also considered as were values and preferences about self-administered DMPA-SC through literature review and a global survey (*2*). Self-administered DMPA-SC was found to be acceptable, easy to use, and preferable to provider administration. Convenience, accessibility, ease of administration, and privacy and confidentiality were important in choosing self-administration. Potential barriers included fear of needles, fear of incorrect administration, and preference for seeing a health care provider. WHO noted insufficient evidence to assess values and preferences from some subgroups, including persons of different age groups. Data on health care providers’ perspectives were limited (*2*).

The 2019 published systematic review (*4*) included two U.S. RCTs that reported implementation outcomes (*6*,*7*). Participants in both studies were provided brief instructional sessions using information from the DMPA-SC package insert, after which they were able to self-administer DMPA-SC. In one of the studies, 97% of participants reported that it was easy to administer the injection at 12 months, and 87% reported high satisfaction with self-administration at 12 months, which was similar to that for provider-administration (92%) (*7*). An additional U.S. study on self-administered DMPA-SC implementation during the COVID-19 pandemic found that 37% of contacted DMPA-IM patients were interested in self-administration of DMPA-SC, and 58% of interested persons reported that they initiated self-administration (*8*). Reasons for not initiating the approach included deciding not to self-administer, moving away, and pharmacy and insurance barriers (*8*).

## Recommendation for Self-Administration of DMPA-SC

CDC adopted the WHO recommendation for self-administered DMPA-SC, which was guided by evidence that the practice increases contraceptive continuation and has equivalent rates of pregnancy, side effects, and associated adverse events compared with provider-administration. The new U.S. SPR recommendation states that self-administered DMPA-SC should be made available as an additional approach to deliver injectable contraception (Box).

BOX Update to U.S. Selected Practice Recommendations for Contraceptive Use*

**New recommendation**

- Self-administered subcutaneous depot medroxyprogesterone acetate (DMPA-SC) should be made available as an additional approach to deliver injectable contraception.

**Comments and evidence summary**

- Self-administered DMPA-SC is a user-controlled method that has the potential to improve contraceptive access and increase reproductive autonomy.
- Self-administered DMPA-SC should be made available as an additional approach; provider-administered DMPA should remain available.
- Self-administered DMPA-SC should be offered in the context of shared decision-making, with a focus on patient preferences and access to the full range of contraceptive methods.
- Existing recommendations in the U.S. Medical Eligibility Criteria for Contraceptive Use and U.S. Selected Practice Recommendations for Contraceptive Use for provider-administered DMPA also apply to self-administered DMPA-SC.
- As with provider-administered DMPA, no routine follow-up is required; however, the patient should be encouraged to contact a health care provider at any time 1) to discuss side effects or other problems, 2) if there is a desire to change the method being used (including requesting provider-administered DMPA), or 3) if there are questions or concerns around re-injection.
- A systematic review and meta-analysis of three randomized controlled trials (RCTs) and three prospective cohort studies compared self-administration of DMPA-SC with provider-administered DMPA-SC or DMPA-IM.^†^
  - Higher rates of continuation were observed with self-administration compared with provider-administration (pooled relative risk [RR] = 1.27, 95% confidence interval [CI] = 1.16–1.39 for three RCTs and pooled RR = 1.18, 95% CI = 1.10–1.26 for three cohort studies).
  - Pregnancy rates were low and did not differ between self-administered and provider-administered groups (four studies).
  - Two studies found higher rates of injection site reactions with self-administered DMPA-SC compared with provider-administered DMPA-IM, and two studies found no differences.
  - No other side effects or adverse events were increased with self-administered DMPA-SC.

* https://www.cdc.gov/reproductivehealth/contraception/mmwr/spr/summary.html

† Kennedy CE, Yeh PT, Gaffield ML, Brady M, Narasimhan M. Self-administration of injectable contraception: a systematic review and meta-analysis. BMJ Glob Health 2019;4:e001350.

## Discussion

Self-administered DMPA-SC might improve access to contraception by removing barriers, such as in-person visits to a health care provider, while promoting empowerment through self-care. As with the WHO recommendation, CDC emphasized that self-administered DMPA-SC should be made available as an additional approach; provider-administered DMPA should remain available. Self-administered DMPA-SC should be offered in a noncoercive, person-centered, and equitable manner, as part of access to the full range of contraceptive methods.

Self-administered DMPA-SC is an option for anyone eligible to use provider-administered DMPA, including adolescents, and the U.S. MEC can be used to assess medical eligibility for DMPA-SC use^§§^^,^^¶¶^ (*3*). Recommendations for initiation, follow-up, and reinjection intervals for self-administered DMPA-SC are the same as those for provider-administered DMPA (*1*). Repeat DMPA injections should be provided every 3 months (13 weeks); the repeat DMPA injection can be given up to 2 weeks late (15 weeks from the last injection) without requiring additional contraceptive protection (*1*). Although the FDA label states that DMPA-SC is only to be administered by a health care professional, health care providers might prescribe an FDA-approved drug for off-label use (including administering a drug in a different way, such as self-administration) when medically indicated, as determined by the health care provider, for their patient.***^,^^†††^ Resources for implementing self-administration of DMPA-SC have been developed by several organizations^§§§^^,^^¶¶¶^^,^**** (*8*). Critical implementation elements to consider include instruction (e.g., in-person or through telemedicine) on self-injection and sharps disposal; access to follow-up care for questions or to switch to provider-administration or another contraceptive method; reinjection reminders; and administrative issues, such as ordering, billing, and reimbursement. Availability of self-administered DMPA-SC expands options for pregnancy prevention and enhances reproductive autonomy when offered in a noncoercive manner through a shared decision-making process between patients and their health care providers, with a focus on patient preferences and equitable access to the full range of contraceptive methods.

**Summary**

**What is already known about this topic?**

Subcutaneous depot medroxyprogesterone acetate (DMPA-SC) is an FDA-approved injectable contraceptive method available in the United States.

**What is added by this report?**

CDC updated recommendations in the U.S. Selected Practice Recommendations for Contraceptive Use to state that self-administered DMPA-SC should be made available as an additional approach to deliver injectable contraception.

**What are the implications for public health practice?**

Self-administration of DMPA-SC is safe and effective and might expand access to a user-controlled contraceptive method. This option should be offered in a noncoercive manner through shared decision-making, with a focus on patient preferences and equitable access to the full range of contraceptive methods, including provider-administered DMPA.

## Footnotes

*
https://www.accessdata.fda.gov/drugsatfda_docs/label/2020/021583s033s034lbl.pdf

†
https://www.cdc.gov/nchs/products/databriefs/db388.htm

§
https://www.cdc.gov/nchs/data/nhsr/nhsr086.pdf

¶ “Self-administration” and “self-injection” include injection by the DMPA-SC user or by someone who is not the user’s health care provider, such as a family or community member.

**
https://www.acog.org/clinical-information/policy-and-position-statements/position-statements/2019/opposition-to-coercive-contraception-practices-and-policies

†† Studies in the systematic review were identified by searching the PubMed, CINAHL, LILACS, and EMBASE databases through September 2018. Meta-analyses were conducted when multiple studies reported the same outcome. WHO used the Grading of Recommendations Assessment, Development and Evaluation approach for recommendation development (full evidence tables can be found at https://apps.who.int/iris/bitstream/handle/10665/325487/WHO-RHR-19.13-eng.pdf?ua).

§§ The recommendations refer to contraceptive methods being used for contraceptive purposes; the recommendations do not consider the use of contraceptive methods for treatment of medical conditions because the eligibility in these situations might differ.

¶¶
https://www.cdc.gov/reproductivehealth/contraception/pdf/summary-chart-us-medical-eligibility-criteria_508tagged.pdf

*** FDA labeling states that DMPA-SC is only to be administered by a health care professional. Therefore, self-administration of DMPA-SC is considered “off-label.”

†††
https://www.fda.gov/patients/learn-about-expanded-access-and-other-treatment-options/understanding-unapproved-use-approved-drugs-label

§§§
https://www.nationalfamilyplanning.org/file/documents---service-delivery-tools/NFPRHA---Depo-SQ-Resource-guide---FINAL-FOR-DISTRIBUTION.pdf

¶¶¶
https://rhntc.org/resources/covid-19-and-family-planning-services-faq

****
https://societyfp.org/wp-content/uploads/2021/02/SFP-Interim-Recommendations-Contraception-and-COVID-19_-02.18.2021.pdf
